# Exploring the material basis and mechanism of action of clinacanthus nutans in treating renal cell carcinoma based on metabolomics and network pharmacology

**DOI:** 10.1097/MD.0000000000035675

**Published:** 2023-10-20

**Authors:** Zhandong Ye, Zhiqiang Fang, Dan Li, Xiaogang Lin, Song Huang

**Affiliations:** a School of Pharmaceutical Science, Guangzhou University of Chinese Medicine, Guangzhou, China; b Department of Pharmacy, Guangdong Second Provincial General Hospital, Guangzhou, China.

**Keywords:** *Clinacanthus nutans*, metabolomics, network pharmacology, renal cell carcinoma

## Abstract

**Background:**

*Clinacanthus nutans* (for abbreviation thereafter) is often used as medicine in the form of fresh juice in the folk to treat many kinds of cancers, including renal cell carcinoma (RCC). It is speculated that its active ingredient may have heat sensitivity, but there are currently no reports on this aspect. Therefore, based on the folk application for fresh juice of *C nutans*, this study used metabonomics and network pharmacology to explore the material basis and mechanism of action of *C nutans* against RCC.

**Methods:**

Firstly, untargeted metabolomics profiling was performed by Liquid chromatography-mass spectrometry and gas chromatography-mass spectrometry to screen the metabolites down-regulated by heat in the extract of *C nutans*. Secondly, we collected the targets of metabolites in the Swiss Target Prediction platform. In addition, the targets of RCC were obtained in the GeneCards database. The “component-target-disease” network was established by Cytoscape3.9.0 software. Then we constructed a protein-protein interaction network in the STRING network platform to screen core targets. The gene ontology and kyoto encyclopedia of genes and genomes enrichment analysis of core targets were carried out to predict the relevant pathway of *C nutans* in the treatment of RCC. Finally, the molecular docking verification of the core targets were carried out.

**Results:**

In this study, 35 potential active ingredients and 125 potential targets were obtained. And the core targets were Cellular tumor antigen p53, Signal transducer and activator of transcription 3, and so on. Then, 48 biological processes, 30 cell components, and 36 molecular functions were obtained by gene ontology enrichment analysis. Besides, 44 pathways were obtained by Kyoto encyclopedia of genes and genomes enrichment analysis, including Pathway in cancer, PI3K-Akt signal pathway, P53 signal pathway, and so on. The docking model between the core target and its corresponding components was stable.

**Conclusion:**

This research is based on the folk application of *C nutans*, showed its potential active ingredients by metabonomics, and predicted the potential mechanism of *C nutans* in the treatment of RCC by network pharmacology. It provides new references for follow-up research and new drug development.

## 1. Introduction

*Clinacanthus nutans*, is a herbaceous plant of the Acanthaceae family, and its leaves are mostly used as medicine.^[1]^ It has a good reputation for cancer treatment among Southeast Asian people and is even called “sambung nyawa” by the Indonesian people, which means life-saving grass.^[2]^ Thailand has listed it as a basic healthcare medicinal plant. In addition, the acute toxicity study of different polar solvent extracts of *C nutans* including ethanol, methanol, and water showed that *C nutans* was safe.^[3–6]^ All kinds of signs show that the herb has the potential to be developed into an anticancer drug. Modern research shows that it has many biological activities such as antitumor, anti-inflammatory, antioxidant, and so on.^[7]^ Most folk people take it orally as medicine in the form of fresh juice,^[8]^ which is used to treat liver cancer, nephritis, kidney cancer, and other diseases. However, the antitumor active ingredients and mechanism of *C nutans* have not been clearly reported.

Renal cell carcinoma (RCC) is a common malignant tumor in the urinary system, which is characterized by strong invasion and high risk of metastasis. In recent years, the incidence rate of RCC has continued to rise in most countries and regions.^[9]^ The onset of RCC is hidden, and it is often in the late stage of cancer when relevant symptoms occur. According to the SEER database of NIH in the United States,^[10]^ with the continuous progress of modern medical technology, although the 5-year survival rate of renal cell carcinoma has been improved, the prognosis of patients with advanced renal cell carcinoma is still not optimistic, and the 5-year survival rate of patients with metastatic renal cell carcinoma is only 12%. The comprehensive treatment based on drug treatment is very important for the improvement of its prognosis.^[11]^ Therefore, the development of new drugs for RCC is very urgent.

Due to the huge advantages of multi-component drugs in the treatment of chronic and complex diseases such as cancer through synergistic effects, traditional Chinese medicine has gradually become a research hotspot.^[12]^ Many unique folk herbs have achieved good results in treating various diseases.^[13]^ However, as the result of the lack of clinical and scientific research, it is difficult to carry out effective development and application without the support of scientific data.^[14]^

Metabolomics is widely used in the study of natural medicine due to its wide coverage and high accuracy, which enables efficient qualitative and quantitative analysis of components.^[15–17]^ Besides, nature medicine act synergistically through multiple components, therefore the mechanisms involved are very complex. Network pharmacology can explore the overall relationship between active ingredients of nature medicine and diseases by establishing a network that connects components and diseases with targets as transition points.^[18–20]^

## 2. Materials and methods

### 2.1. Source of medicinal materials

The Clinacanthus nutans used in this project was introduced from Wuzhi Mountain in Hainan to Huolu Mountain Planting Base in Guangzhou. It was collected and identified as *Clinacanthus nutans* by Professor Chen Jiannan of Guangzhou University of Traditional Chinese Medicine and its samples were stored in the New Chinese Medicine Development Laboratory of Guangzhou University of Traditional Chinese Medicine.

### 2.2. Sample preparation

The fresh leaves of *C nutans* were freeze-dried and then superfine crushed. After 30 minutes of ultrasonic extraction with 10 times of dichloromethane, the filtrate is naturally volatilized in the fume hood for 12 hours, and then freeze-dried for 24 hours. Finally, extract was obtained, and it was named as freeze-dried sample, which was prepared 3 times and numbered F-1, F-2, F-3; Take a certain amount of extract and heat it at 105°C for 6 hours, and name it as heated sample. Repeat the preparation for 3 times, numbered H-1, H-2, H-3. The samples were stored at 4°C.

### 2.3. Liquid chromatography-mass spectrometry (LC-MS) analysis

#### 2.3.1. Sample submission for inspection.

Take a certain amount of the freeze-dried sample and the heated sample in the centrifuge tube and seal them for storage. Put the centrifuge tube into dry ice and send it to Chengqi Medical (Shenzhen) Technology Co., Ltd. for testing.

#### 2.3.2. Sample pretreatment.

Add precooled methanol-acetonitrile-water solution to the sample, ultrasonic for 30 minutes, 14,000 g centrifugation for 20 minutes, take the supernatant for vacuum drying, add acetonitrile water solution for redissolution, 14,000 g centrifugation for 15 minutes, take the supernatant for analysis.

#### 2.3.3. Chromatography-mass spectrometry analysis.

The sample injection volume is 2.0 μL. The column temperature is 40°C and the flow rate is 0.4 mL/minutes; Mobile phase A is 25 mM ammonium acetate −0.5% formic acid water; Mobile phase B is methanol. Mobile phase gradient downward: 0 to 0.5 minutes, 5%B; 0.5 to 10 minutes, 5% to 100%B; 10.0 to 12.0 minutes, 100%B; 12.0 to 12.1 minutes, 100% to 5%B; 12.1 to 16 minutes, 5%B; QC samples are inserted into the samples to monitor the stability of the system. AB Triple TOF 6600 mass spectrometer was used for sample data collection.

#### 2.3.4. Data analysis.

The metabolite structure identification is carried out in the metabolome database of new life plants in the Chinese family. Finally, the data are processed and analyzed, including univariate statistical analysis, multidimensional statistical analysis, differential metabolite screening, etc.

### 2.4. Gas chromatography-mass spectrometry (GC-MS) analysis

#### 2.4.1. Sample pretreatment.

Take a certain amount of freeze-dried sample and heated sample samples and add dichloromethane to make the final concentration of 1.0 mg/mL, ultrasound for 30 minutes, and pass 0.45μm microporous membrane, filtrate analysis.

#### 2.4.2. Chromatography-mass spectrometry analysis.

The sample injection volume is 1.0 μL. The flow rate is 1.0 mL/minutes. The heating procedure is as follows: 0 to 2 minutes, 60°C; 2 to 10 minutes, 60°C to 180%°C; 10.0 to 15.0 minutes, 180%°C; 15.0 to 32.5 minutes, 250%°C. After running at 300°C for 5 minutes, conduct the next sample injection, and insert the QC sample into the sample. The mass spectrometer is used for full scan mode scanning of m/z 50 to 550, and the mass spectrum is collected at 70eV.

#### 2.4.3. Data analysis.

Identify the name and molecular weight of the compound in the NIST library. Then conduct data analysis on the Metware Cloud platform (https://cloud.metware.cn/), including univariate statistical analysis, multidimensional statistical analysis, differential metabolite screening, etc.

### 2.5. Network pharmacological analysis

#### 2.5.1. Collection of ingredients.

Collect the down-regulated metabolites in the above metabolomics and get their SMILES formula in the PubChem database (https://pubchem.ncbi.nlm.nih.gov/). The oral availability and drug-like properties were investigated on the Swiss ADME platform (http://www.swissadme.ch/).^[21]^ Collect ingredients that are “high” in gastrointestinal absorption and “yes” in Lipinski.

#### 2.5.2. Collection of active ingredient targets of *C nutans.*

We predict the targets of the components that meet the conditions in the Swiss Target Prediction platform (http://www.swisstargetprediction.ch/),^[22]^ and take “probability > 0.1” as the screening criteria. Compare and determine the targets in the Uniprot database (https://www.uniprot.org/), and obtain the targets of the ingredients of *C nutans* at last.

#### 2.5.3. Collection of RCC disease targets.

Collect potential targets related to renal cell carcinoma by the keywords “Renal cell carcinoma”, “RCC”, and “Kidney cancer” in the Gene Cards database (https://www.genecards.org/). According to the correlation score, the top 5% of the targets are selected as the disease targets of RCC.

#### 2.5.4. Construction of “components-targets-disease” network.

The Venn Diagram is established in the Bioinformatics platform (http://www.bioinformatics.com.cn/) to obtain the intersection targets of *C nutans* and RCC, and import it into Cytoscape3.9.0 software (https://cytoscape.org/)^[23]^ for visualization processing to build a “component-target-disease” network.

#### 2.5.5. Protein-protein interaction (PPI) network.

Introduce intersection genes into the STRING network platform (https://cn.string-db.org/),^[24]^ the species option is “Homesapiens,” the interaction score ≥ 0.4, the PPI network is established, and the target point of network interruption is deleted. After the core targets are obtained by screening with the median of Degree, Betweenness Centrality, and Closeness Centrality, the PPI network is reestablished under the same conditions, and the results are visualized.

#### 2.5.6. Gene ontology (GO) enrichment analysis.

The core targets are imported into the Metascape platform (https://metascape.org/).^[25]^ The species option is “Homosapiens,” which is used for enrichment analysis of biological processes, cell components, and molecular functions. Import the analysis results into the Glue GO^[26]^ plug-in, and conduct cluster analysis under the conditions of *P* value ≤ .01, Kappa score = 0.12. The results are made into a bubble chart according to the size of *P* value.

#### 2.5.7. Kyoto encyclopedia of genes and genomes (KEGG) enrichment analysis.

The core targets are imported into the Metascape platform, and the species option is “Homesapiens.” The KEGG enrichment analysis is carried out under the condition of *P* value ≤ .01 and the minimum number of overlapping genes is 12. Import the analysis results into the Glue GO plug-in, and conduct cluster analysis under the conditions of *P* value ≤ .01, Kappa score = 0.32. The results are made into a bubble chart according to the size of *P* value.

#### 2.5.8. Molecular docking.

Collect the core targets in PPI and get its corresponding components. Collect the structure file of the targets in the PDB database (https://www.rcsb.org/)^[27]^ and obtain the 3D structure of the components from PubChem. Vina software^[28]^ is used for molecular docking to obtain the lowest binding energy. The docking results are visualized through Discovery Studio software.

## 3. Results

### 3.1. The results of LC-MS analysis

#### 3.1.1. Overview of metabolites from the extract of *C nutans.*

**Figure 1.**
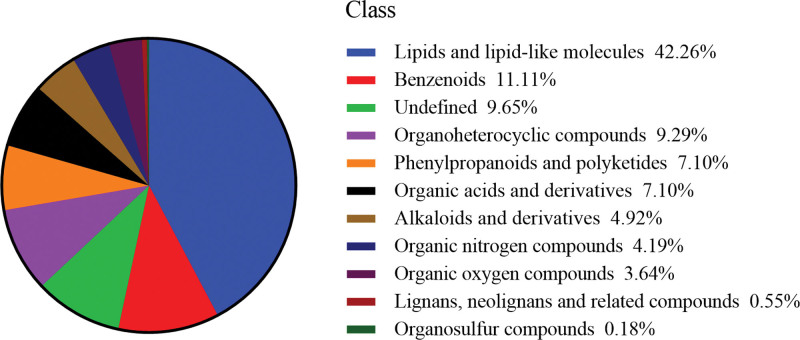
Metabolites from the extract of CN. CN = *Clinacanthus nutans*.

A total of 549 metabolites were identified from the samples of freeze-dried sample and heated sample. It includes 232 kinds of lipids and lipid-like molecules, 61 kinds of benzenoids, 51 kinds of organoheterocyclic compounds, 39 kinds of phenylpropanoids and polyketides, 39 kinds of organic acids and derivatives, 27 kinds of alkaloids and derivatives, 23 kinds of organic nitrogen compounds, 20 kinds of organic oxygen compounds, 3 kinds of lignans, neolignans and related compounds, 1 kind of organosulfur compounds, and 53 kinds of other compounds (Fig. 1).

#### 3.1.2. Principal component analysis (PCA).

**Figure 2.**
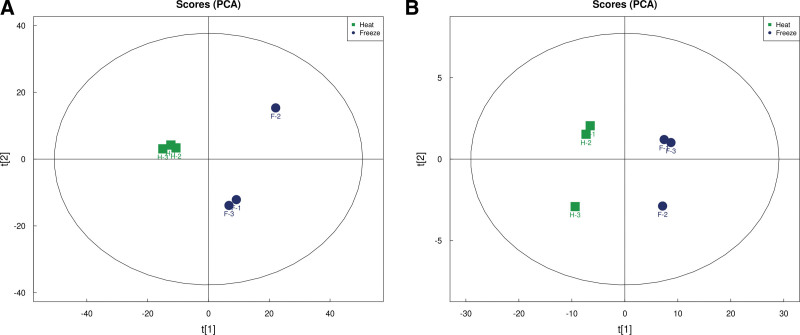
PCA diagram of CN by LC-MS. CN = *Clinacanthus nutans*, LC-MS = liquid chromatography-mass spectrometry, PCA = principal component analysis.

PCA analysis can visually observe whether the analyzed samples are grouped, check the occurrence of abnormal samples and observe the classification information of samples. From Figure 2, it can be found that the samples of the freeze-dried sample and the heated sample are obviously separated on both sides by the Y axis, indicating that the samples of the freeze-dried sample and the heated sample have obviously different metabolites.

#### 3.1.3. Screening of differential metabolites.

**Figure 3.**
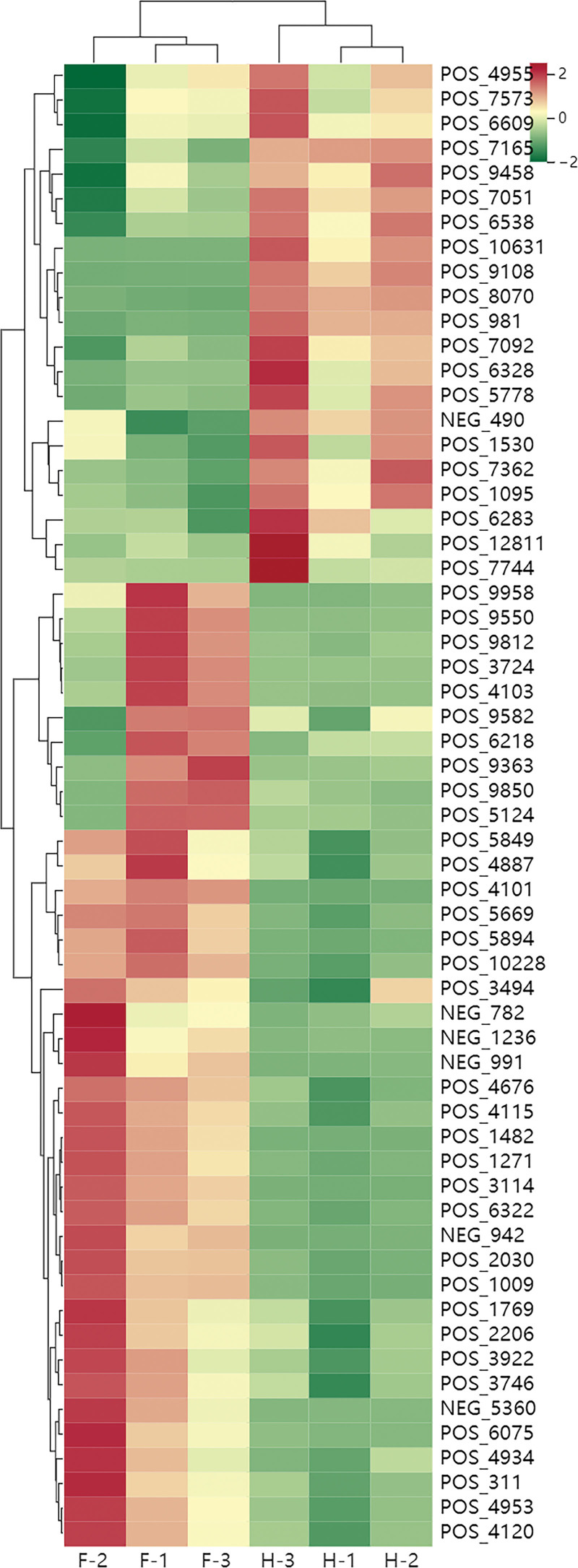
Different metabolites by cluster heat map.

Combined with univariate analysis and OPLS-DA analysis, metabolites were screened with FC>1.6 or FC<0.625 and VIP>0.8, and a total of 60 different metabolites were obtained. Among them, there are 27 kinds of lipids and lipid-like molecules, 6 kinds of benzenoids, 6 kinds of organoheterocyclic compounds, 4 kinds of organic acids and derivatives, 4 kinds of organic nitrogen compounds, 3 kinds of phenylpropanoids and polyketones, 1 kind of alkaloids and derivatives, 1 kind of organic oxygen compounds, and 8 kinds of other compounds. The differential metabolites were analyzed by cluster heat map (Fig. 3). Compared with the freeze-dried sample, 39 components were down-regulated and 21 components were up-regulated in the heated sample.

### 3.2. The results of GC-MS analysis

#### 3.2.1. Overview of metabolites from the extract of *C nutans.*

The samples of freeze-dried sample and heated sample were tested. Thirty-one metabolites were identified. It includes 12 kinds of lipids and lipid-like molecules, 5 kinds of benzenoids, 3 kinds of organoheterocyclic compounds, 3 kinds of organic silicon compounds, 1 kind of alkaloids and derivatives, and 7 kinds of other compounds.

#### 3.2.2. PCA.

**Figure 4.**
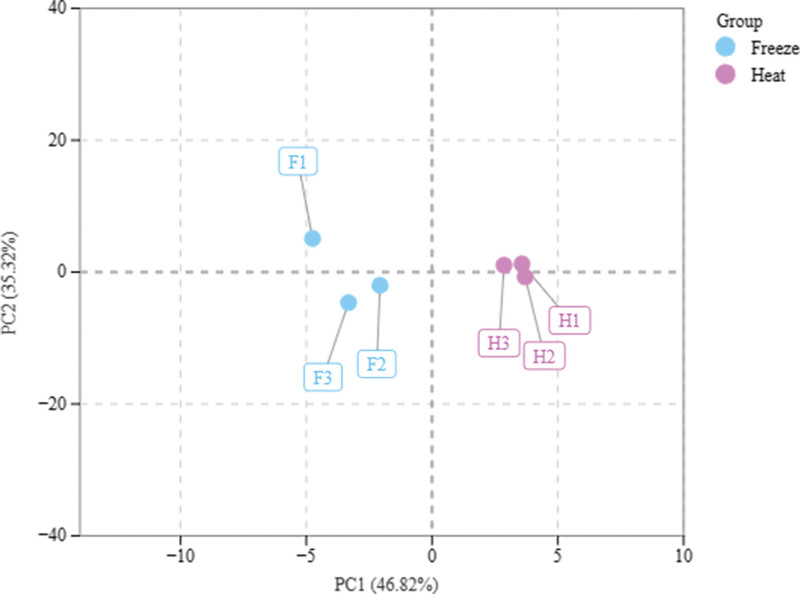
PCA diagram of CN by GC-MS. CN = *Clinacanthus nutans*, GC-MS = gas chromatography-mass spectrometry, PCA = principal component analysis.

Carry out PCA analysis on the Metware Cloud platform. From Figure 4, it can be found that the samples of the freeze-dried sample and the heated sample are obviously separated on both sides by the Y axis, indicating that the samples of the freeze-dried sample and the heated sample have obvious different metabolites.

#### 3.2.3. Screening of differential metabolites.

**Figure 5.**
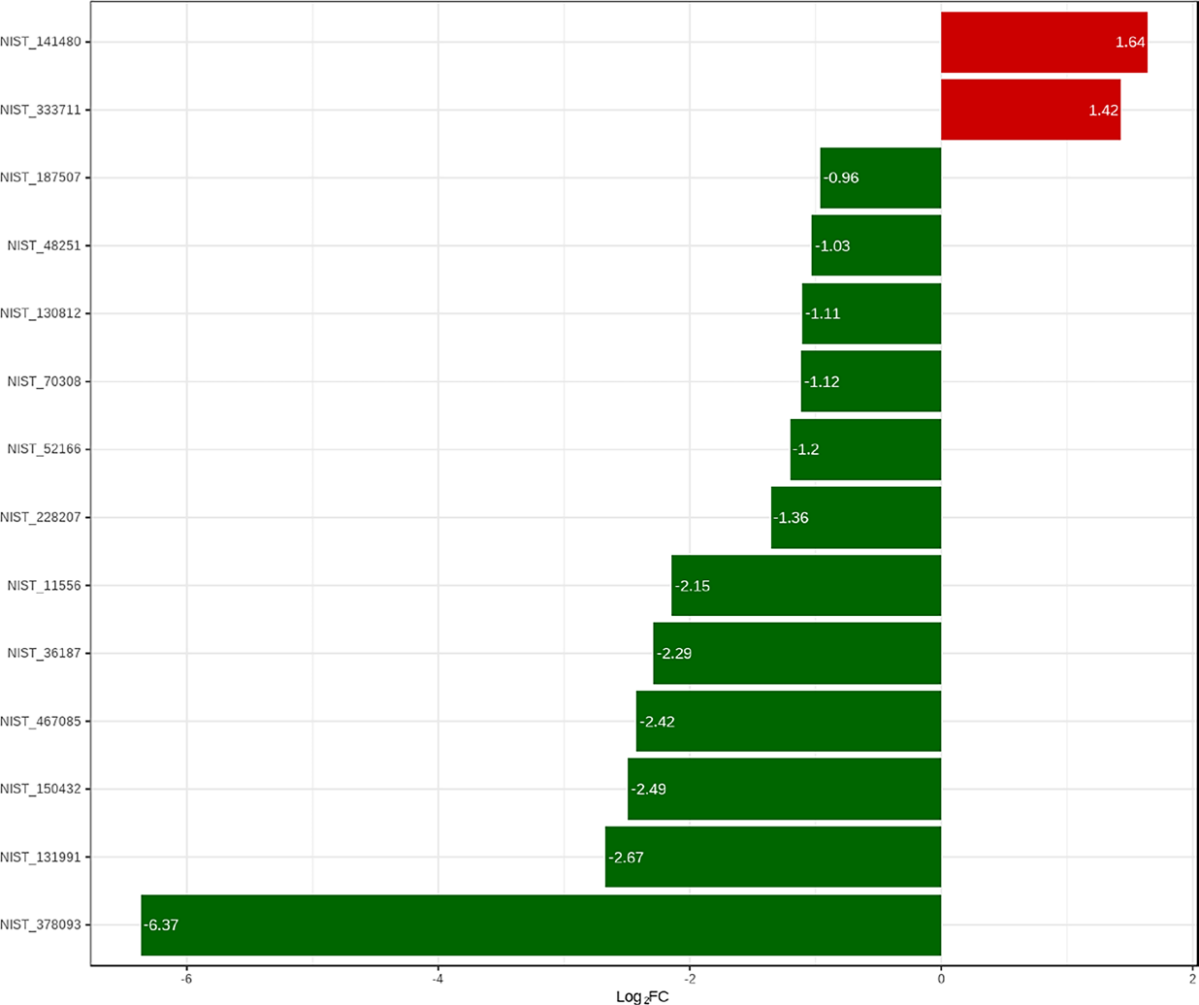
Different metabolites by bar chart.

Combined with univariate analysis and OPLS-DA analysis, metabolites were screened with FC>1.6 or FC<0.625 and VIP>0.8, and a total of 14 different metabolites were obtained. Display the difference multiple of different metabolites with a bar chart (Fig. 5). Compared with the freeze-dried sample, the heated sample down-regulated 12 components and up-regulated 2 components.

### 3.3. The results of network pharmacological analysis

#### 3.3.1. Collection of ingredients.

In the metabonomics study, compared with the freeze-dried sample, there were 51 down-regulated components in the heated sample. Thirty-five active ingredients were obtained by screening under the conditions of gastrointestinal absorption and drug-likeness, which were name *C nutans*_(1–35)(Table 1).

**Table 1:** Components information for network pharmacology.

ID	Pubchem ID	Compound	Molecular formula	m/z
CN_1	23815359	Spiro [11-oxatricyclo [4.4.1.01, 6] undeca-3, 8-diene-10, 3’-2, 4-dioxatricyclo [7.3.1.05, 13] trideca-1 (12), 5, 7, 9 (13), 10-pentaene]-2, 5, 7-triol	C_20_H_16_O_6_	391.0495
CN_2	3126	2-Aminooctadecane-1, 3-diol	C_18_H_39_NO_2_	302.3032
CN_3	122121	Phytosphingosine	C_18_H_39_NO_3_	318.3009
CN_4	248575	2-Aminooctadecane-1, 3, 4-triol	C_18_H_39_NO_3_	318.2987
CN_5	53941620	8-(2-Oxo-5-pent-2-enylcyclopent-3-en-1-yl) octanoic acid	C_18_H_28_O_3_	291.1947
CN_6	5374699	Jasmolone	C_11_H_16_O_2_	181.1214
CN_7	72965	Ailanthone	C_20_H_24_O_7_	399.1402
CN_8	10215903	Apo-12’-capsorubinal	C_25_H_34_O_3_	383.2584
CN_9	3787294	N-(2-Hydroxyethyl) icosanamide	C_22_H_45_NO_2_	356.3524
CN_10	139291938	4-[(3R, 5R, 8R, 9S, 12S, 14S, 17R)-3, 12-Dihydroxy-10, 13-dimethyl-2, 3, 4, 5, 6, 7, 8, 9, 11, 12, 14, 15, 16, 17-tetradecahydro-1H-cyclopenta[a]phenanthren-17-yl] pentanoic acid	C_24_H_40_O_4_	375.2851
CN_11	44559173	Corchorifatty acid F	C_18_H_32_O_5_	327.2160
CN_12	936	Nicotinamide	C_6_H_6_N_2_O	123.0544
CN_13	23786427	(5R)-5-Hydroxy-1-(4-hydroxy-3-methoxyphenyl) tetradecan-3-one	C_21_H_34_O_4_	351.2524
CN_14	5283266	Persin	C_23_H_40_O_4_	381.2990
CN_15	5282944	9-Hydroxy-10E, 12Z-octadecadienoic acid	C_18_H_32_O_3_	295.2275
CN_16	23844024	18, 19-Dihydroxykaur-16-en-6-one	C_20_H_30_O_3_	319.2255
CN_17	5673	Ethyl eburnamenine-14-carboxylate	C_22_H_26_N_2_O_2_	351.2150
CN_18	11746327	5-(6-Methyl-6-hydroxyoctyl) furan-2 (5H)-one	C_13_H_22_O_3_	209.1527
CN_19	138404421	(5S, 6E, 8S, 9Z, 13S, 14R)-5-Hydroxy-8-methoxy-5, 9, 13, 14-tetramethyl-1-oxacyclotetradeca-6, 9-dien-2-one	C_18_H_30_O_4_	311.2188
CN_20	75368758	8-[5-(Acetyloxy)-3-methylpentyl] octahydro-4, 4, 8a-trimethyl-7-methylene-2 (1H)-naphthalenone	C_22_H_36_O_3_	349.2663
CN_21	13918478	(E)-5-(1, 2, 4a, 5-Tetramethyl-7-oxo-3, 4, 8, 8a-tetrahydro-2H-naphthalen-1-yl)-3-methylpent-2-enoic acid	C_20_H_30_O_3_	341.2081
CN_22	57509432	16, 17-Dihydroxy-7-kauranone	C_20_H_32_O_3_	303.2335
CN_23	5373082	8-[3-Oxo-2-[(E)-pent-2-enyl] cyclopenten-1-yl] octanoic acid	C_18_H_28_O_3_	293.2018
CN_24	5321018	Atractylenolide I	C_15_H_18_O_2_	231.1372
CN_25	452967	Steviol	C_20_H_30_O_3_	319.2229
CN_26	10465	Heptadecanoic acid	C_20_H_30_O_3_	319.2229
CN_27	91692121	N-Chloroacetyl-3, 6, 9, 12-tetraoxapentadec-14-yn-1-amine	C_13_H_22_ClNO_5_	307.1187
CN_28	538234	Acetic acid, 2-acetoxymethyl-1, 2, 3-trimethylbutyl ester	C_12_H_22_O_4_	230.1518
CN_29	10911	Cyclohexasiloxane, dodecamethyl-	C_12_H_36_O_6_Si_6_	444.1127
CN_30	5385014	1, 16-Cyclocorynan-17-oic acid, 19, 20-didehydro-, methyl ester, (16S, 19E)-	C_20_H_22_N_2_O_2_	322.1681
CN_31	571996	Chloroethyl 2-hexyl ether	C_8_H_17_ClO	164.0968
CN_31	5363512	E-2-Methyl-3-tetradecen-1-ol acetate	C_17_H_32_O_2_	268.2402
CN_33	7311	2, 4-Di-tert-butylphenol	C_14_H_22_O	206.1671
CN_34	91719722	Phthalic acid, hex-3-yl isobutyl ester	C_18_H_26_O_4_	306.1831
CN_35	66540	1, 2-Benzenedicarboxylic acid, butyl octyl ester	C_20_H_30_O_4_	334.2144

CN = *Clinacanthus nutans*.

#### 3.3.2. Analysis of the intersection gene between *C nutans* and RCC.

**Figure 6.**
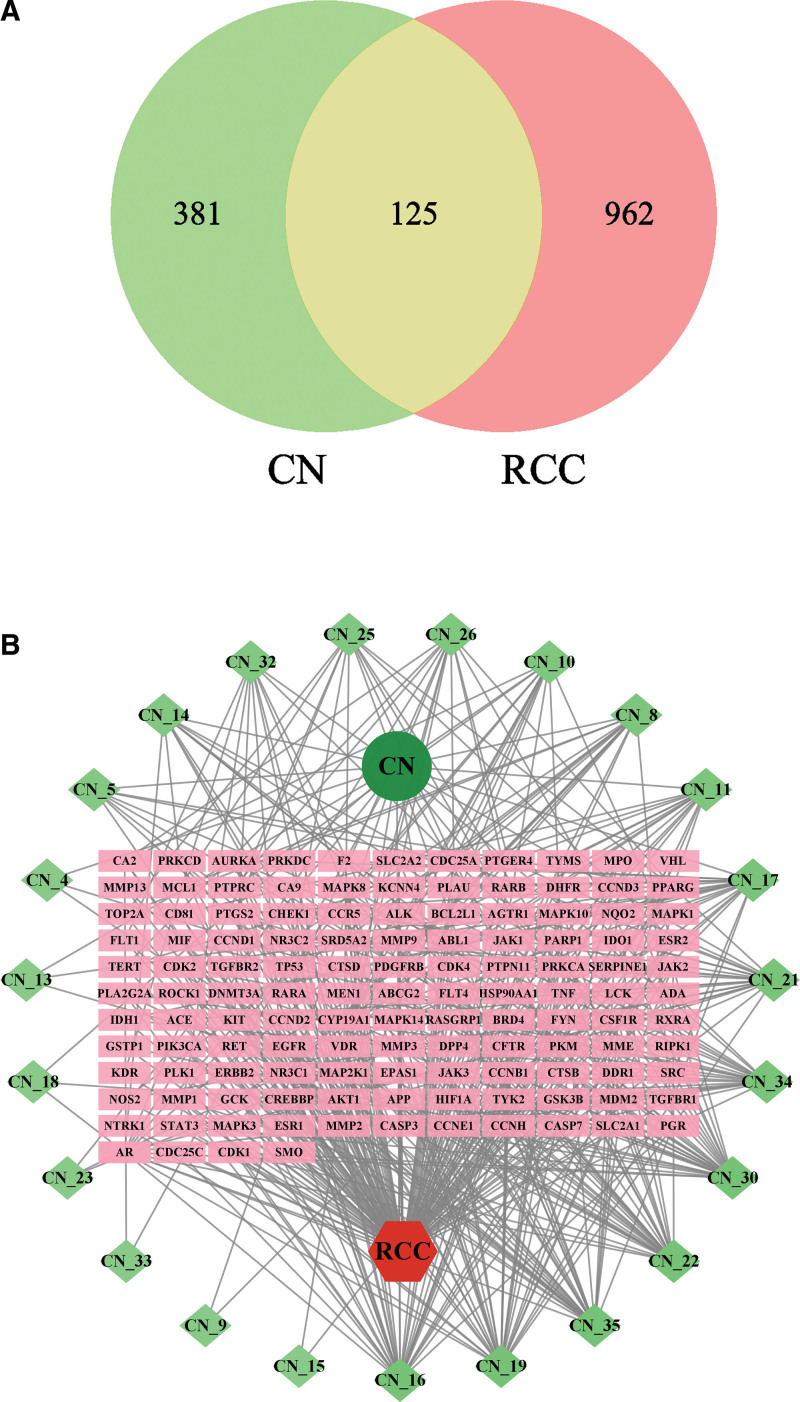
The network of CN and RCC (A) the venn of CN and RCC; (B) the network of “components-target-disease”. CN = *Clinacanthus nutans*, RCC = renal cell carcinoma.

Through prediction and screening on the Swiss Target Prediction platform, 506 potential targets of *C nutans* were obtained at last. A total of 21,744 targets related to RCC were retrieved in the Gene Cards database, and 1087 targets with the top 5% correlation score were collected. A total of 125 intersection targets were obtained after the intersection with the targets of *C nutans*, and the “components-targets-disease” network was established and visualized (Fig. 6).

#### 3.3.3. PPI analysis.

**Figure 7.**
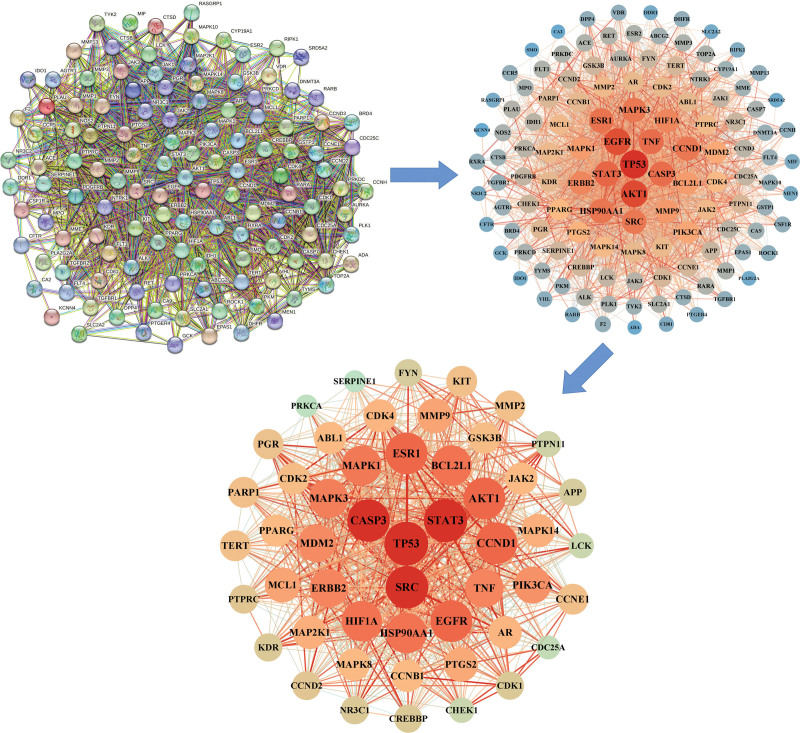
PPI core network diagram of common targets. PPI = protein-protein interaction.

In order to explore the protein-protein interaction between the intersection targets, a PPI network was built through the STRING platform. Finally, the PPI network diagram with 51 nodes and 888 functions was obtained (Fig. 7). The larger and redder the node in the figure, the more important it is in the network. It can be seen from the figure that cellular tumor antigen p53 (TP53), signal transducer and activator of transcription 3 (STAT3), SRC, and CASP3 may be the core targets of *C nutans* in the treatment of RCC.

#### 3.3.4. GO enrichment analysis.

**Figure 8.**
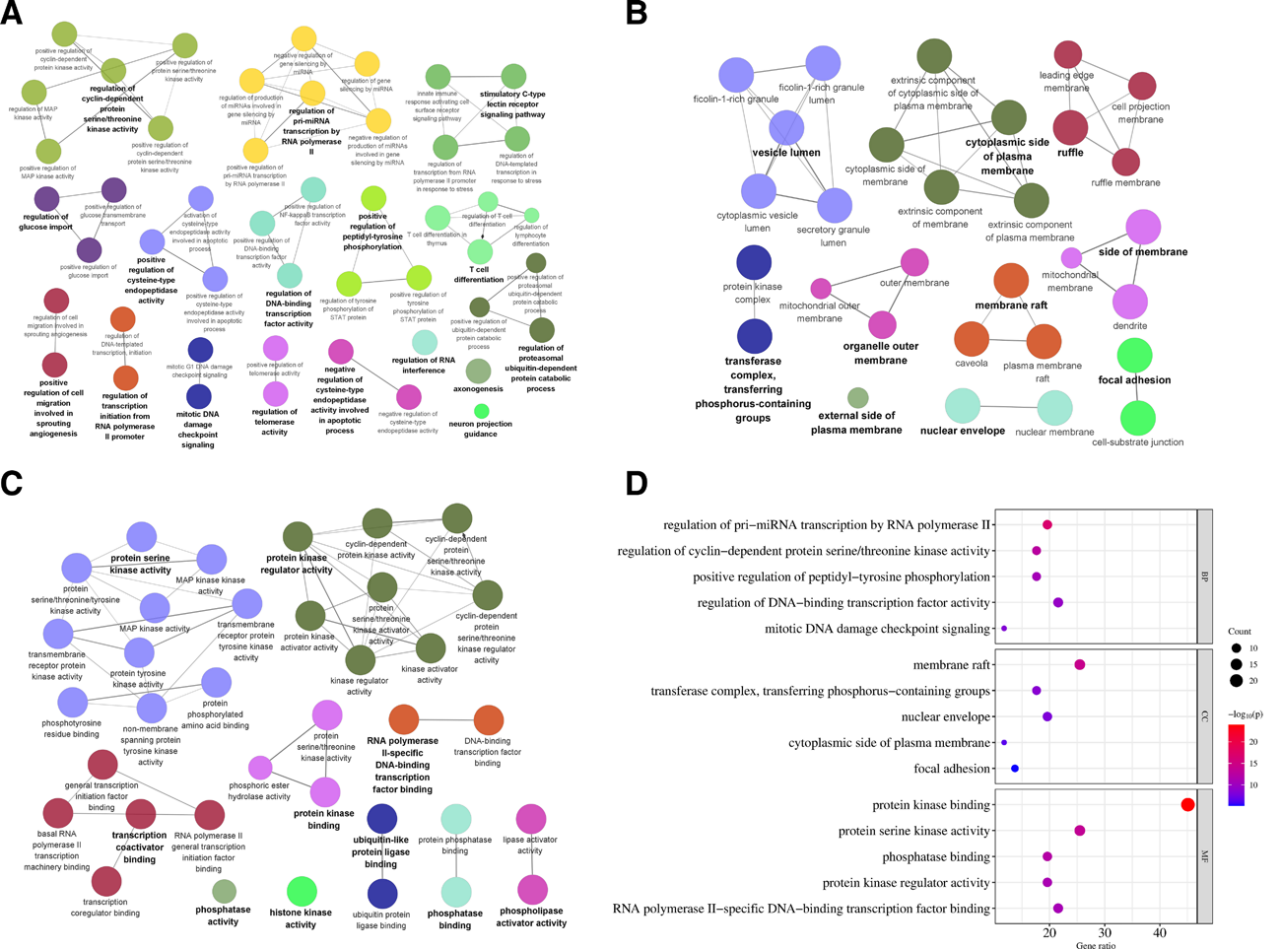
GO enrichment analysis (A) cluster diagram of BP, (B) cluster diagram of CC, (C) cluster diagram of MF, and (D) bubble chart of GO analysis. BP = biological processes, CC = cell components, GO = gene ontology, MF = molecular functions.

The results of GO analysis showed that 48 biological processes, 30 cell components, and 36 molecular functions were obtained by co-enrichment. The biological processes involved include regulation of pri-miRNA transcription by RNA polymerase II, regulation of cyclin-dependent protein serine/threonine kinase activity, positive regulation of peptide-tyrosine phosphorylation, and so on; The cell components involved include membrane rafts, transferase complexes, transferring phosphorus-containing groups, nuclear membranes, etc; The molecular functions involved include protein kinase binding, protein serine kinase activity, phosphatase binding, etc. Sort the *P* value from small to large, and filter the first 5 items to draw a bubble chart. The redder the color is, the smaller the *P* value is, and the higher the correlation is (Fig. 8).

#### 3.3.5. KEGG enrichment analysis.

**Figure 9.**
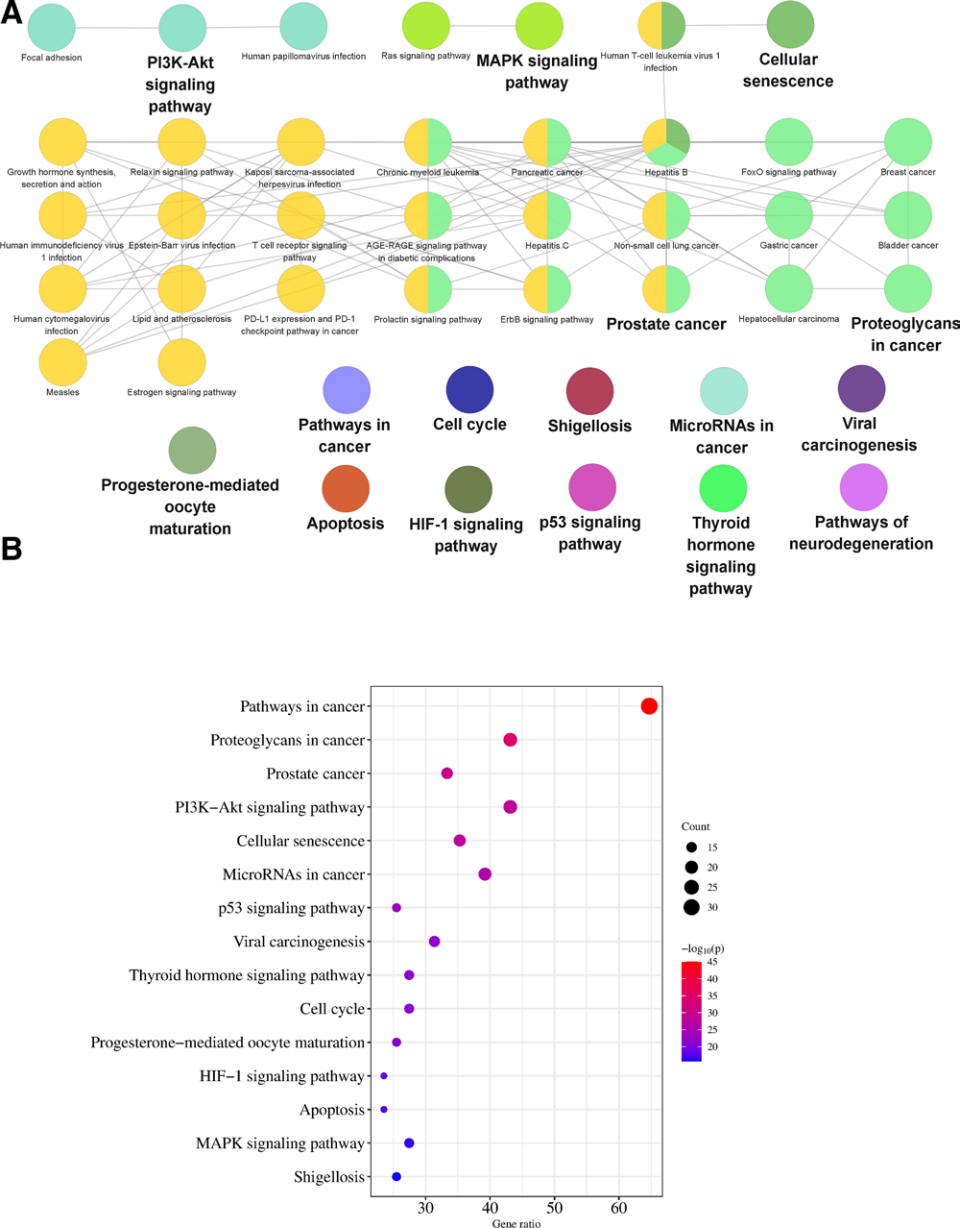
GO enrichment analysis (A) cluster analysis of KEGG, (B) bubble chart of KEGG analysis. GO = gene ontology, KEGG = Kyoto encyclopedia of genes and genomes.

KEGG enrichment analysis of core genes was carried out through the Metascape platform and Glue GO plug-in. Finally, 44 channels were obtained. It mainly includes pathway in cancer, cell cycle, apoptosis, etc. It also includes PI3K-Akt signal pathway, p53 signal pathway, MAPK signal pathway, and other signal pathways. The bubble chart is drawn according to the first 15 key channels of *P* value screening. The redder the color is, the smaller the *P* value is, and the higher the correlation is (Fig. 9).

#### 3.3.6. Molecular docking results.

**Figure 10.**
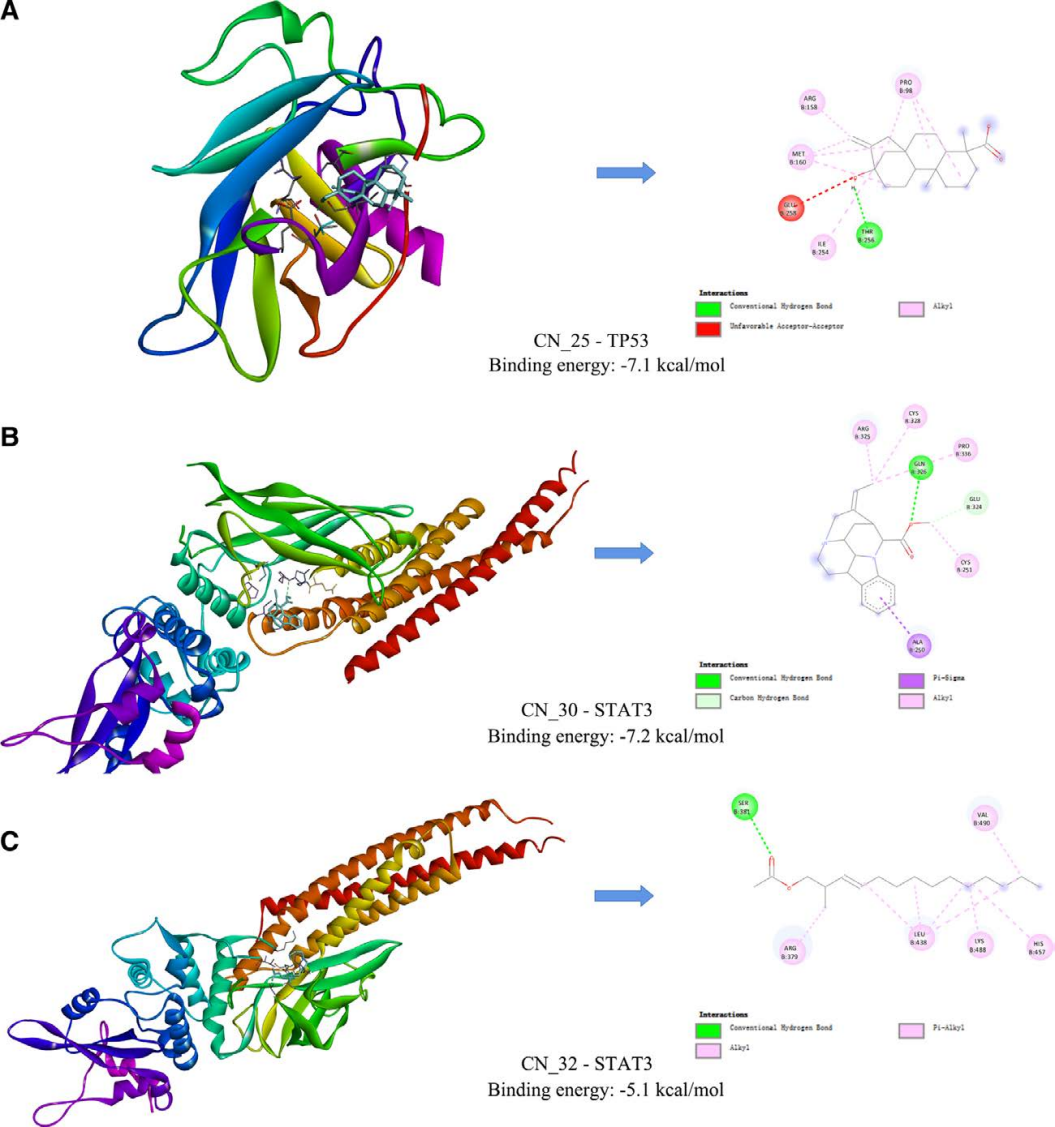
The results of molecular docking.

Select the core target TP53 (PDB ID: 2VUK) and STAT3 (PDB ID: 6TLC) with the highest degree value obtained in the network pharmacology research to establish the molecular docking model with their corresponding components. The binding activity of components and target proteins is expressed in the form of binding energy.^[29]^ When the minimum binding energy is <0 kcal/mol, components and proteins can combine spontaneously; When the minimum binding energy is less than −5.0 kcal/mol, the 2 have good binding activity. The molecular docking model simulated in this study shows that the components and receptors bind well (Fig. 10). It shows that the core target obtained in network pharmacology research can be effectively combined with its corresponding active components.

## 4. Discussion

At present, the mainstream of the research on *C nutans* is the flavonoid components such as Schaftoside, vitexin, orientin,^[30]^ but this does not mean that some unusual components can be ignored. Although these flavonoids have been reported to have antitumor activity, they are widely found in many plants^[31–33]^ and are not unique to *C nutans*. According to the use of fresh extract of *C nutans* as medicine, it is speculated that the active ingredients of *C nutans* against RCC may have the characteristics of easy decomposition or volatilization when heated. Combined with some studies, the dichloromethane part of *C nutans* is potential to have the best antitumor activity.^[34]^

Metabolomics is the qualitative and quantitative analysis of biological metabolites under certain conditions.^[35]^ It has the advantages of wide coverage and high accuracy.^[36,37]^ It mainly uses high-resolution mass spectrometry to identify and analyze the chemical components in traditional Chinese medicine. By comparing the differences between samples, important differences are screened out from huge data.^[38,39]^ LC-MS and GC-MS are widely used because of their high sensitivity and good reproducibility. LC-MS is mainly used for metabolites with high boiling point and poor thermal stability, while GC-MS is mainly used for metabolites with low boiling point and low polarity. The metabonomics study using LC-MS and GC-MS simultaneously can effectively improve the integrity of identifying all differential metabolites.^[40,41]^ Therefore, based on folk application, this study screened the metabolites related to drug efficacy in the dichloromethane extract of *C nutans* by metabonomics.^[42,43]^

Drugs exert their effects mainly in the form of regulating biological networks.^[44]^ However, due to the wide variety of components of traditional Chinese medicine, the complexity of the effect of traditional Chinese medicine is far higher than that of single drugs.^[45]^ Therefore, the method of network pharmacology is adopted to take into account the complex situation of multiple targets and synergistic effects of traditional Chinese medicine when it exerts its efficacy, so as to explore the mechanism of *C nutans* in treating RCC.^[46,47]^

According to the results of network pharmacology, PI3K-Akt signal pathway and p53 signal pathway may be the potential pathway of *C nutans* in the treatment of RCC. Inhibiting the activation of PI3K/Akt/m TOR signal pathway can inhibit the progress of cell cycle,^[48]^ and also regulate the expression of Bax and Bcl-2, thus inducing the process of cell apoptosis.^[49,50]^ It has been reported that the growth of renal cell carcinoma cells can also be inhibited by regulating the PI3K/mTOR/p53 signal pathway.^[51]^

TP53 gene, also known as p53, is an important tumor suppressor gene, which can activate p21, Bax and other downstream target genes to interfere with cell cycle, apoptosis, etc., and then inhibit the development of cancer.^[52]^ However, in almost all renal cell carcinomas, p53 exists as wild-type, and its signal pathway will lose its anticancer function due to tissue or disease specificity. The abnormal condition of VHL, HIF, MDM2, MDM4, and other genes linked to the development of renal cell carcinoma is closely related to the disappearance of its anticancer function. Even in some renal cell carcinomas with p53 mutations, p53 also shows a cancer-promoting effect.^[53]^ Therefore, the role of p53 in renal cell carcinoma needs further study. STAT3 is critical for cancer progression by regulating tumor cell survival, proliferation, and angiogenesis.^[54]^ Besides, STAT3 instability and degradation can effectively inhibit the development of RCC.^[55]^

## 5. Conclusion

In summary, according to the folk application of *C nutans*, this study screened the heat-sensitive ingredients by untargeted metabolomics profiling. These ingredients were used for network pharmacology and molecular docking research. The research results showed that these components have a good effect on anti-RCC. Therefore, these ingredients are the potential active ingredients of *C nutans* in anti-RCC. It provides a certain research direction for *C nutans* against RCC, and further experiments will be carried out in the future.

## Author contributions

**Conceptualization:** Zhandong Ye.

**Data curation:** Dan Li.

**Formal analysis:** Zhiqiang Fang, Dan Li, Xiaogang Lin.

**Funding acquisition:** Song Huang.

**Investigation:** Zhiqiang Fang, Song Huang.

**Methodology:** Zhandong Ye.

**Software:** Zhiqiang Fang.

**Validation:** Song Huang.

**Visualization:** Zhandong Ye.

**Writing – original draft:** Zhandong Ye.

**Writing – review & editing:** Song Huang.
